# Notes and News

**Published:** 1891-01-10

**Authors:** 


### Notes and News.

Collected and Communicated.

The Hospital Saturday Fund is now a vigorous national institution; the usefulness of which is increasing every year. The 18th annual meeting of the supporters of the Hospital Saturday and Sunday Fund at Norwich was very largely attended, and was remarkable as being the first which has been attended by ladies. The Mayor of Norwich presided, and said that the nature of the report was exceedingly satisfactory. The receipts in the aggregate were greater than any that had gone before, and they had at their command a balance largely in excess of last year. This satisfactory state of affairs was due, in some measure, to the efforts of Mrs. Dakin, and other ladies of Norwich, who had organised a street collection and gathered, in one day, the sum of £187 1s. 3d.

The little people at the Foundling Hospital kept their Christmas with beef and plum-pudding, sweets and oranges, to which, according to long honoured custom here, was added a glass of ginger wine. For many years a kind anonymous friend of the hospital has made a point of sending a bright new penny for each child. This is placed for a surprise under the mug standing beside each plate. "Truth" had sent everyone a toy, and these arranged together in tempting display, were shown to the children, who have other treats in store, and will then receive them. All lessons were suspended for the day, and when dinner was over the children amused themselves to their hearts' content until bed-time. At all hospitals, Christmas Day is made the occasion of festivity and rejoicing. The wards are prettily decorated with wreaths and festoons of holly and evergreens, which cover the walls, and sometimes even the cots are adorned. At Guy's Hospital this year a number of students, dressers, and doctors gave the patients a nigger minstrel entertainment, and went the round of the wards, accompanied by a number of visitors and improvised a variety entertainment with bones, banjo, and piano, which was greatly appreciated by the patients. At Bartholomew's there was no special entertainment. The patients, however, had a fête day so far as Christmas fare is concerned, and for just this once in a way were allowed to smoke.

There is to be a new sick hospital for Dundee. Dr. Littlejohn, the medical officer for the Board of Supervision, has minutely examined the plans of the proposed building, and has expressed himself as very highly satisfied with the general principles on which they has been prepared. Dr. Littlejohn then visited the site which has been selected for the hospital, and after going over the ground he congratulated the members of the Board on their having acquired so suitable a piece of ground. In its proximity to the poor-house and general surroundings a better site could not be desired.

The Governors of the Sheffield General Infirmary have granted the means to enable two of their medical men, who recently visited Berlin for the purpose of making inquiries as to Professor Koch's discoveries, to carry on their investigation. With the sum given—£100—they will improve their microscope and get such requirements as will enable them to study the life history of the tuberculous bacillus and to acquire the fluid invented by Professor Koch for its destruction. The staff will then be able to treat with all necessary safeguards the many cases of tuberculous diseases which are constantly applying to the Infirmary for relief.

One backward glance at the hospital work of the year. The year opened well with Mr. Peter Reid's donation of £100,000 for a Metropolitan Convalescent Home, and on the first day of the year the Hartley Memorial Wing of the Sunderland Infirmary was opened. A Committee was appointed at Birmingham to inquire into the alleged abuse of hospitals; the Nexborough Montague Hospital was opened on January 30th. In February died Sir William Gull; the Redruth Hospital for Women was opened; also the Victoria Infirmary, Glasgow; and the new Workhouse Infirmary at Coventry. The foundation stone of the Hull Victoria Hospital for Children was laid. In March Mr. Cadbury, of Birmingham, gave £30,000 towards a Convalescent Home for Children, and Lady Howard de Walden gave £10,000 to the West Kent Hospital; Mr. Wainwright was elected treasurer of St. Thomas's; Mr. Gladstone opened the new Medical College at Guy's; and the foundation stone of the new wing of the Hospital for Sick Children, Great Ormond Street, was laid.

In April the West Ham Hospital was opened, and the Hospitals Association announced the success of its scheme for supplying the metropolis with ambulance stations. The Lords' Committee on Hospitals commenced its sittings. In May the foundation-stone of the new buildings of the London Hospital was laid, an Infectious Hospital was opened at Derby, and the Ipswich Hospital secured its chapel. In June the branch hospital at the docks of the Seamen's Hospital Society was opened by the Prince of Wales; the first general meeting of the Hospital Saturday Fund was held; Paisley Sick Hospital was opened; Mr. B. E. Fletcher undertook to present the Norfolk and Norwich Hospital with a convalescent home. In July died Sir Edwin Chadwick and Sir Richard Wallace; the All Saints' Convalescent Home for Children was opened; also the Woolwich Cottage Hospital and the Eye Hospital, Swansea. In August the Stephen Cottage Hospital at Dufftown was opened, and the foundation-stone of the Birmingham Ear and Throat Hospital was laid. In September the Felixstowe Convalescent Home was opened; also one at St. Anne's-on-the-Sea. A new wing was added to the Wigan Infirmary; Sunderland procured an Infectious Hospital, and Thirsk a Cottage Hospital. In October the Bradford Children's Hospital was opened; also the Mansfield Hospital. In November the Liverpool Royal Infirmary was opened; also a Cottage Hospital at Kirkcaldy, and a Convalescent Home at Torquay.

The following vacancies are announced this week, the amount of salary in each case being given after the name of the institution :—

**Resident Medical Officers.**

Brompton Hospital, House Physicians.
East London Hospital for Children, House Surgeon—Board, &c.
Kent and Canterbury Hospital, Assistant House Surgeon and Dispenser—£50, board, &c.
Lancashire County Asylum, Pathological Assistant Medical Officer—£200, rooms, &c.
Important Public College, Medical Office and Bursar—£200, board, &c.
Denbighshire Infirmary, House Surgeon—£85, board, &c.
Norfolk and Norwich Hospital, House Surgeon—£100, board, &c.

**Non-Resident Medical Officers.**

Lismore Union, Medical Officer—£117.
Marylebone General Dispensary, Honorary Surgeon.
London Hospital Medical College, Assistant Demonstrator of Anatomy—£90.

---

Christmas day was kept at King's College Hospital and at St. Thomas's without any special demonstration, but the annual Christmas Free Entertainment at the former institution will be held some time in January. At the Royal Free Hospital a Christmas service was held, at which all the convalescent patients were present. Liberal Christmas fare was provided, and all through the day the patients were allowed to receive their relatives and friends.

---

A fever hospital has just been completed at Portobello. It is built in two parts, which are joined by a covered way. In the front portion are the doctor's room, nurse's room, and extra accommodation for patients, if required. The main building contains a male and female ward, with complete lavatory accommodation attached to each ward, and there is a bath-room, with a travelling bath. The cost of the buildings was about £1,500.

---

Mr. Egerton Burnett has offered to give the town of Wellington the site and building for a Cottage Hospital, provided that the inhabitants of the town would keep up the cost of maintenance of the ten beds the cottage would accommodate. To consider this generous proposal, the leading residents assembled in the Town Hall on Thursday evening, and discussed the question in all its bearings. Mr. C. H. Fox, president of the Taunton Hospital, reminded those present that in 1884 a meeting had been held to discuss the same question, and it had then been decided that it was more desirable that patients from the town should be taken to Taunton, and for this purpose an ambulance was provided. The case was now the same as then. It had never been a question of money, but of expediency. He added that patients from Wellington, at Taunton, cost a little over £200 a-year, and having been intimately associated with the hospital for five years, he knew how kindly everyone was looked after. He hoped that whatever was done, the number of Wellington subscribers to the hospital would not be lessened, but the more they could do for the poor the better, and if the new scheme were approved he would put down his name as a subscriber. The Rev. G. W. Humphreys, in proposing that the meeting be adjourned to receive the report of the Committee, added that, as one who saw a great deal of sickness in the town, he rejoiced very much in the movement. There were a large number of cases, such as rheumatic fever and lung diseases, and to such the hospital would be a great boon. He hoped the residents would be prompted to support the movement and raise subsciptions. Mr. F. H. Fox seconded, remarking that the question required a good deal of consideration, and time was needed to make inquiries. A vote of thanks to the Chairman concluded the proceedings.

Dr. Strange, of Worcester, died very suddenly on Monday last. He took his degree at Edinburgh in 1839, went on the honorary medical staff of the Worcester Infirmary in 1861, and was elected President of the British Medical Association in 1882. Dr. Strange was a clever writer, and contributed to many of the leading periodicals. His loss will be severely felt in Worcester, where he was much respected.

---

There is to be a set of three dances under the patronage of Princess Christian, at the Portman Rooms, in aid of the North-West London Hospital, Kentish Town Road. These dances have been admirably organised, and promise to be most successful. It has been determined by a capable Committee, consisting of Mrs. George Herring, Dr. Walter Sibley, and Mr. A. Craske, that the North-West London dances are to be conducted on much the same principles as the Portman ones. The Hungarian Band has been engaged for the series. The first dance will take place on Wednesday, January 21st. Among the ladies interested in these dances are: Lady Carden, Mrs. Jeune, Mrs. Blandford, Mrs. Burdett, Mrs. Lionel Thomson, Mrs. Robert Webster, Mrs. William Flower, Mrs. Graily Hewitt, and Mrs. St. Aubyn. The subscription for the three dances will be one guinea, and for single tickets for one dance half-a-guinea. For further particulars application should be made to Mrs. George Herring, 6, Park Crescent, W.

---

The parishioners of Strath met last week for the purpose of opposing the proceedings of the Mackinnon Memorial Hospital Committee. The Committee, with the assistance of others interested in the scheme, collected a sum of about £900, all of which was given on the express understanding that the hospital was to be built at Broadford, where Dr. Mackinnon was born, and where he laboured as a minister for the long period of thirty years. The Committee had afterwards, without any valid reason, changed their minds, and fixed Isleornsay, in the parish of Sleat, as the site for the hospital. The Rev. Dr. Mackay presided, and the following resolutions were moved, and carried unanimously: 1. That this meeting strongly protests against such change of site. 2. That this meeting use every means in their power to have the funds now in the hands of the Committee applied to the purpose for which they were subscribed, namely, a cottage hospital at Broadford to the memory of the late Dr. Mackinnon. 3. That copies of these resolutions be sent to Lord Macdonald, Sir William Mackinnon, and to the secretary of the Mackinnon Memorial Hospital. The proceedings then terminated.

---

On December 17th, Mr. Craven, J.P., presided at a meeting of the Hull Hospital Sunday Committee. Councillor T. J. Smith, Hon. Secretary, submitted his report, which stated that this was the seventeenth year of collection, and showed that pretty nearly the same amount had been collected as in the past year, notwithstanding the inauspicious weather. The total amount received was £624 14s. 11d., against £639 2s. 6d. in 1889 and £603 14s. 3d. in 1888. The Committee thanked the ex-Mayor, and added that the balance in the hands of Messrs. Pease and Sons, hon. treasurers, was 4s. 2d., which, added to the collections made during the year, made £624 19s. 1d. The expenses amounted to £30 19s. 1d., leaving for distribution £594. This it was proposed to divide as follows: Hull Royal Infirmary, £468; Hull Dispensary, £60; Victoria Children's Hospital, £50; Homœopathic Dispensary, £15—in all £593; leaving a balance of £1, against 4s. 2d. last year. The chairman moved the adoption of the report, and, in doing so, regretted that the collections had not reached £1,000. Alderman Seaton seconded the motion, which was supported by the Rev. Dr. Lambert and Alderman Sherburn. The report was adopted, and it was decided that a meeting of the General Committee be held early in the New Year.

